# Supplementary material for: Machine Learning Accelerates Crystallization for Structure Determination
Source: Angew Chem Int Ed Engl. 2026 May 4;65(25):e1218503. doi: 10.1002/anie.1218503 (PMC13266958; doi:10.1002/anie.1218503)

checkCIF/PLATON report

Structure factors have been supplied for datablock(s) lcz044

THIS REPORT IS FOR GUIDANCE ONLY. IF USED AS PART OF A REVIEW PROCEDURE FOR PUBLICATION, IT SHOULD NOT REPLACE THE EXPERTISE OF AN EXPERIENCED CRYSTALLOGRAPHIC REFEREE.

No syntax errors found. CIF dictionary Interpreting this report

Datablock: lcz044

Bond precision: C-C = 0.0128 A Wavelength=1.54184

Cell:	a=12.8099(1)	b=22.8396(2)	c=13.1736(1)
	alpha=90	beta=114.948(1)	gamma=90
Temperature:	100 K		

	Calculated	Reported
Volume	3494.61(6)	3494.60(6)
Space group	P 21/c	P 1 21/c 1
Hall group	-P 2ybc	-P 2ybc
Moiety formula	C15 H3 Ag3 F18 N6, C15 H12 O2	C15 H3 Ag3 F18 N6, C15 H12 O2
Sum formula	C30 H15 Ag3 F18 N6 O2	C30 H15 Ag3 F18 N6 O2
Mr	1157.09	1157.09
Dx,g cm-3	2.199	2.199
Z	4	4
Mu (mm-1)	14.642	14.642
F000	2224.0	2224.0
F000’	2234.55	
h,k,lmax	16,29,16	16,28,16
Nref	7515	7211
Tmin,Tmax	0.074,0.231	0.523,1.000
Tmin’	0.005	

Correction method= # Reported T Limits: Tmin=0.523 Tmax=1.000
AbsCorr = MULTI-SCAN

Data completeness= 0.960 Theta(max)= 78.456

R(reflections)= 0.0519(6264) wR2(reflections)= 0.1386(7211)

S = 1.083 Npar= 560

The following ALERTS were generated. Each ALERT has the format
test-name_ALERT_alert-type_alert-level.
Click on the hyperlinks for more details of the test.

Alert level C

PLAT213_ALERT_2_C Atom F2 has ADP max/min Ratio ..... 3.3 prolat
PLAT213_ALERT_2_C Atom F00N has ADP max/min Ratio ..... 3.1 prolat
PLAT213_ALERT_2_C Atom F00Q has ADP max/min Ratio ..... 3.2 prolat
PLAT234_ALERT_4_C Large Hirshfeld Difference F1A --C3 . 0.18 Ang.
PLAT250_ALERT_2_C Large U3/U1 Ratio for <U(i,j)> Tensor(Resd 1) 2.1 Note
PLAT309_ALERT_2_C Single Bonded Oxygen (C-O > 1.3 Ang) .... O00C Check
PLAT342_ALERT_3_C Low Bond Precision on C-C Bonds .... 0.01279 Ang.
PLAT362_ALERT_2_C Short C(sp3)-C(sp2) Bond C00W - C013 . 1.37 Ang.
PLAT906_ALERT_3_C Large K Value in the Analysis of Variance ...... 8.391 Check
PLAT906_ALERT_3_C Large K Value in the Analysis of Variance ...... 2.021 Check
PLAT911_ALERT_3_C Missing FCF Refl Between Thmin & STh/L= 0.600 26 Report
13 0 2, -7 24 5, -8 6 7, -9 2 11, -14 6 11, -13 7 11,
-4 17 11, -12 3 12, -11 3 12, -10 3 12, -12 4 12, -11 4 12,
-10 4 12, -12 5 12, -11 5 12, -12 6 12, -10 2 13, -12 0 14,
-12 1 14, -8 5 14, -9 9 14, -9 10 14, -8 10 14, -5 1 15,
-7 7 15, -7 8 15,
PLAT977_ALERT_2_C Check Negative Difference Density on H01A . -0.71 eA-3
PLAT977_ALERT_2_C Check Negative Difference Density on H01D . -0.38 eA-3

Alert level G

PLAT002_ALERT_2_G Number of Distance or Angle Restraints on AtSite 7 Note
PLAT003_ALERT_2_G Number of Uiso or U(i,j) Restrained non-H-Atoms 7 Report
PLAT083_ALERT_2_G SHELXL Second Parameter in WGHT Unusually Large 69.47 Why ?
PLAT142_ALERT_4_G s.u. on b - Axis Small or Missing .... 0.00020 Ang.
PLAT143_ALERT_4_G s.u. on c - Axis Small or Missing .... 0.00010 Ang.
PLAT176_ALERT_4_G The CIF-Embedded .res File Contains SADI Records 3 Report
PLAT186_ALERT_4_G The CIF-Embedded .res File Contains ISOR Records 2 Report
PLAT242_ALERT_2_G Low 'MainMol' Ueq as Compared to Neighbors of C3 Check
PLAT242_ALERT_2_G Low 'MainMol' Ueq as Compared to Neighbors of C012 Check
PLAT242_ALERT_2_G Low 'MainMol' Ueq as Compared to Neighbors of C01D Check
PLAT242_ALERT_2_G Low 'MainMol' Ueq as Compared to Neighbors of C01E Check
PLAT242_ALERT_2_G Low 'MainMol' Ueq as Compared to Neighbors of C01F Check
PLAT242_ALERT_2_G Low 'MainMol' Ueq as Compared to Neighbors of C01G Check
PLAT301_ALERT_3_G Main Residue Disorder ....(Resd 1) 7% Note
PLAT434_ALERT_2_G Short Inter HL..HL Contact F004 ..F007 . 2.82 Ang.
1-x,2-y,1-z = 3_676 Check
PLAT434_ALERT_2_G Short Inter HL..HL Contact F008 ..F00A . 2.84 Ang.
1-x,1/2+y,3/2-z = 2_656 Check
PLAT434_ALERT_2_G Short Inter HL..HL Contact F00B ..F1A . 2.65 Ang.
1+x,3/2-y,1/2+z = 4_676 Check
PLAT720_ALERT_4_G Number of Unusual/Non-Standard Labels .... 70 Note

```
              Ag01    Ag02    Ag03    F004    F005    F006    F007    F008
              O009    F00A    F00B    O00C    F00D    F00E    F00F    F00G
              N00H    N00I    N00J    N00K    N00L    N00M    F00N    F00O
              F00Q    F00S    C00T    H00T    C00U    C00V    H00V    C00W
              C00X    H00X    C00Y    C00Z    C010    C011    H011    C012
              C013    H01A    H01B    C015    C016    H016    C017    C018
              C019    H019    C01A    C01B    H01C    C01C    H01D    C01D
              C01E    C01F    C01G    C01H    H01H    C01I    H01I    C01J
              H01J    C01K    C01M    H01M    C01N    H01N
PLAT860_ALERT_3_G Number of Least-Squares Restraints ............         45 Note
PLAT883_ALERT_1_G Absent Datum for _atom_sites_solution_primary ..     Please Do !
PLAT912_ALERT_4_G Missing # of FCF Reflections Above STh/L=  0.600        278 Note
PLAT933_ALERT_2_G Number of HKL-OMIT Records in Embedded .res File         14 Note
              -11  4 12, -12  5 12, -11  5 12, -12  4 12, -12  6 12, -12  3 12,
              -14  7 11, -14  6 11, -11  3 12, -10  4 12, -13  7 11, -14  8 11,
              -10  3 12,  -9  2 11,
PLAT941_ALERT_3_G Average HKL Measurement Multiplicity ..........        2.5 Low
PLAT969_ALERT_5_G The 'Henn et al.' R-Factor-gap value ..........      3.347 Note
              Predicted wR2: Based on SigI**2  4.14 or SHELX Weight 12.80
PLAT978_ALERT_2_G Number C-C Bonds with Positive Residual Density.          0 Info
```

```
   0 ALERT level A = Most likely a serious problem - resolve or explain
   0 ALERT level B = A potentially serious problem, consider carefully
  13 ALERT level C = Check. Ensure it is not caused by an omission or oversight
  25 ALERT level G = General information/check it is not something unexpected

   1 ALERT type 1 CIF construction/syntax error, inconsistent or missing data
  22 ALERT type 2 Indicator that the structure model may be wrong or deficient
   7 ALERT type 3 Indicator that the structure quality may be low
   7 ALERT type 4 Improvement, methodology, query or suggestion
   1 ALERT type 5 Informative message, check
```

Validation response form

Please find below a validation response form (VRF) that can be filled in and pasted into your CIF.

```
# start Validation Reply Form
_vrf_PLAT213_lcz044
;
PROBLEM: Atom F2              has ADP max/min Ratio .....        3.3 prolat
RESPONSE: ...
;
_vrf_PLAT234_lcz044
;
PROBLEM: Large Hirshfeld Difference F1A      --C3       .       0.18 Ang.
RESPONSE: ...
;
_vrf_PLAT250_lcz044
;
PROBLEM: Large U3/U1 Ratio for <U(i,j)> Tensor(Resd    1)        2.1 Note
RESPONSE: ...
;
_vrf_PLAT309_lcz044
;
PROBLEM: Single Bonded Oxygen (C-O > 1.3 Ang) ..........       O00C Check
```

```
RESPONSE: ...
;
_vrf_PLAT342_lcz044
;
PROBLEM: Low Bond Precision on  C-C Bonds ...............    0.01279 Ang.
RESPONSE: ...
;
_vrf_PLAT362_lcz044
;
PROBLEM: Short  C(sp3)-C(sp2) Bond  C00W     - C013     .       1.37 Ang.
RESPONSE: ...
;
_vrf_PLAT906_lcz044
;
PROBLEM: Large K Value in the Analysis of Variance ......      8.391 Check
RESPONSE: ...
;
_vrf_PLAT911_lcz044
;
PROBLEM: Missing FCF Refl Between Thmin & STh/L=    0.600         26 Report
RESPONSE: ...
;
_vrf_PLAT977_lcz044
;
PROBLEM: Check Negative Difference Density on H01A      .      -0.71 eA-3
RESPONSE: ...
;
# end Validation Reply Form
```

It is advisable to attempt to resolve as many as possible of the alerts in all categories. Often the minor alerts point to easily fixed oversights, errors and omissions in your CIF or refinement strategy, so attention to these fine details can be worthwhile. It is up to the individual to critically assess their own results and, if necessary, seek expert advice.

PLATON version of 17/09/2025; check.def file version of 17/09/2025

---

duplicate check

No duplication found

Datablock lcz044 - ellipsoid plot

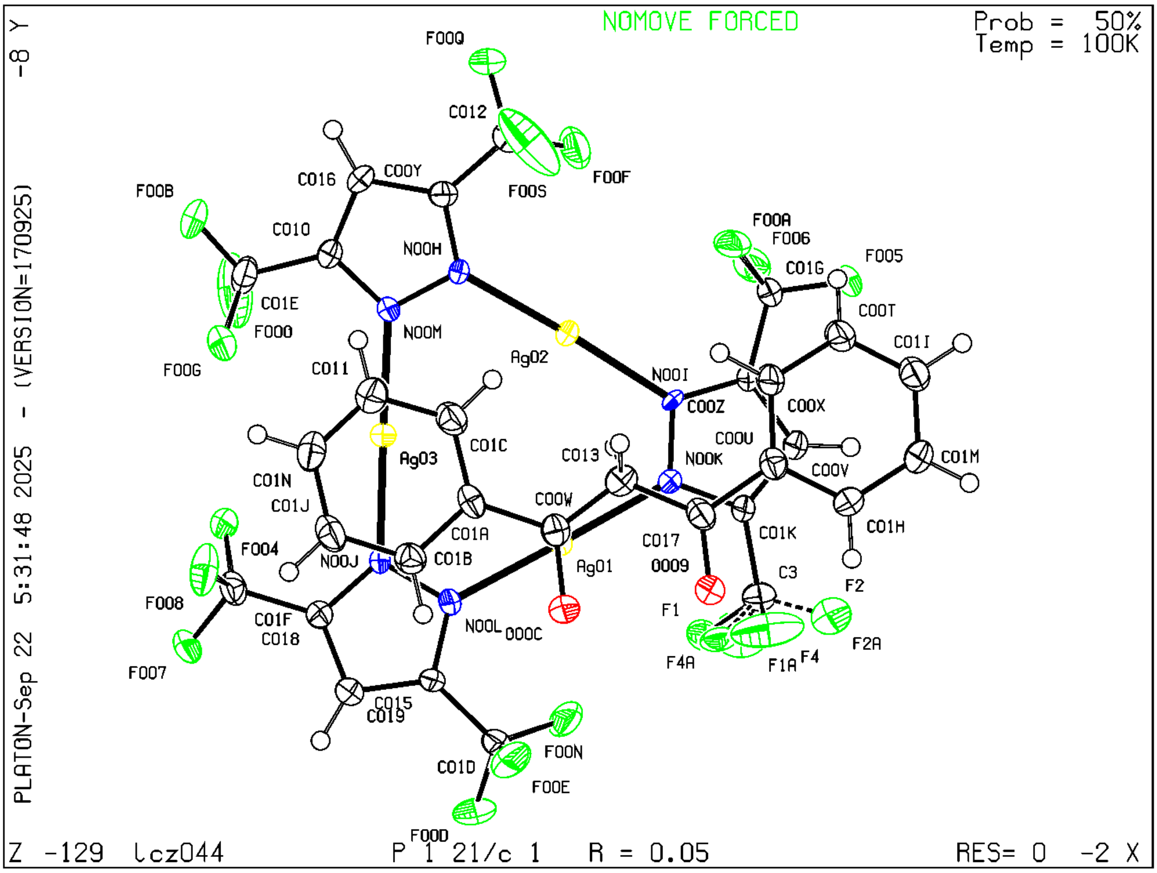

Supplement: Supplementary file 2 — Supporting File 2: anie72462‐sup‐0002‐DataFile.zip. [file ANIE-65-e1218503-s002.zip › supplementary_materials_crystallographic_data submit.zip!/cif_and_checkcif(1-114)/Ag3Pz3_44/Ag3Pz3_44_checkcif.pdf]
